# Editorial: Expanding the Science of Compassion

**DOI:** 10.3389/fpsyg.2021.745799

**Published:** 2021-09-13

**Authors:** Myriam Mongrain, Dacher Keltner, James Kirby

**Affiliations:** ^1^Department of Psychology, York University, Toronto, ON, Canada; ^2^Department of Psychology, University of California, Berkeley, Berkeley, CA, United States; ^3^School of Psychology, University of Queensland, St Lucia, QLD, Australia

**Keywords:** compassion, meditation, contemplative research, evolutionary models of compassion, compassion focused therapy, prosociality, compassion interventions

This special edition examines the physiological, psychological, and environmental conditions facilitating the experience of compassion. This is timely given the global challenges facing our world and the need for collective action. The barriers that inihibit our capacity for goodness are included in this collection, along with numerous applications to implement a compassionate mindset at the individual collective level. This work is inspired by a “sea change” in evolutionary science (see Wilson, 2020) suggesting that a concious evolution toward a more compassionate world is possible. We bring together reknowned researchers from different disciplines to offer novel theoretical concepts, research methods, results, and applications to help transform our future.

The issue is loosely organized along four main themes: (1) Definition of compassion along with ways of measuring it, (2) Brain-based and peripheral biological systems that support this motivational orientation, (3) Applications, methods, and techniques designed to promote compassion at the individual level, and (4) Strategies to help collectives adopt a compassionate stance toward all its members.

## Defining and Measuring Compassion

Gilbert provides a comprehensive explanation of the ontogeny and evolutionary history of compassion. He reviews the necessary personal experiences, the competencies, and physiological support systems necessary for compassion to be expressed toward the self and toward others. He offers the following definition of compassion as “*a sensitivity to suffering in self and others with a commitment to try to alleviate and prevent it*” (Gilbert, 2017).

Mascaro et al. reiterate the definition of compassion as a “benevolent emotional response toward another who is suffering, coupled with the motivation to alleviate their suffering and promote their well-being.” They draw upon an abundant literature across disciplines to provide a coherent rubric of the various ways in which compassion has been measured and tested. They forge important connections between disparate areas of research and provide a thorough overview of the current state of available methods for studying compassion.

## Biological Contributions

In an ambitious paper, Ho et al. offer a brain-based Bayesian inference framework to highlight the information processing pathways involved in “ego preserving biases” that compromise compassionate responding. The juxtaposition of Buddhist concepts onto emerging findings in neuroscience is a tour-de-force. The authors manage to weave in the operations of large functional systems within the brain that hyjack empathic concern toward another's suffering. The role of mind training to bypass ego-biases in situations of interpersonal conflict are incorporated within a neuro functional model.

Self-soothing in the form of self-reassurance has also been mapped onto the brain. The areas underlying the experience of compassion toward the self are believed to stem from the same motivational system underlying compassion toward others. In their study, Kim et al. show that participants high on trait self-criticism show different patterns of brain activation during a self-reassurance task. Their results demonstrate how difficulties in being kind to oneself can be captured in distinct brain processes. This research adds to the physiological underpinnings of compassionate responding.

Miller et al. demonstrate the role of the peripheral nervous system in the expression of compassion. There is a rich literature connecting compassion to parasympathetic activation, including heart rate variability and vagal tone (Porges, 2017; Stellar and Keltner, 2017). Individual differences in these functions emerge early, and Miller et al. show how vagal flexibility in children helps them orient toward another's distress and help support their ability for calm engagement. They also demonstrate the role of the mother's levels of compassion in predicting altruistic action for those children with vagal flexibility. The findings are in support of a “biopsychosocial” model of prosociality.

In their opinion paper, Clapton and Hiskey propose that martial arts can help provide the training for greater sympathovagal balance. They describe how deliberate and controlled movement in mind-body practices can stimulate the parasympathetic nervous system and produce resilience. The various forms of martial arts are said to preserve relational harmony, in part through behavioral synchrony and a compassionate embodiment of conflict resolution.

The capacity for empathic concern and love toward humanity may also be facilated by an abstract thinking style. Fernández et al. found that following the Parisian terrorist attack in 2015, Spanish participants with a more abstract cognitive style reported greater empathic concern toward the victims up to 2 months following the attack. The authors suggest that abstraction skills may help promote collective reactions of compassion by allowing representations of a better future.

## Increasing Compassion Toward Self and Others

“Compassion Focused Therapy” (CFT; https://doi.org/10.3389/fpsyg.2020.586161) targets physiological processes directly involved in the capacity to engage in supportive way with one's own suffering. This therapy aims at long term changes in one's compassionate response by developing the inner ability to feel safe and supported. Because the tools in CFT are varied and target different systems, Gilbert refers to it as a type of *neuropsychophysiotherapy*, one of its kind being theoretically grounded and supported by research.

Petrocchi et al. applied CFT to a sample of treatment-resistant OCD patients and obtained significant reductions in symptoms. These patients had not previously responded to traditional therapies. The authors suggest that the techniques of CFT may have been instrumental in reducing compulsive compensatory behaviors by activating patients' self-soothing capacities.

Many meditative techniques rely on Buddhist notions about the nature of mind and suffering. Ho et al. propose a compassion meditation technique to cultivate attunement to universal suffering. In this process, the authors provide a compelling argument for the benefits accrued through a re-wiring of the brain that allows detachment from ego-related concerns. Further, meditation practice allows for a glimpse of the true nature of mind which is innately compassionate.

Condon and Makransky present other meditation tools under “Sustainable Compassion Training” (SCT). It consists of a number of incremental steps to help respond to others with unconditional care and discernment for right action. The foundation of this model relies on the development of a secure base activating inner qualities of safety, acceptance and love. Guided meditations in SCT are linked throughout the text of this article, and can be applied immediately.

Transmuting our own pain and empathizing with humanity is a quality of mind that may help maintain compassion. Baguley et al. examined the strategies used by health care professionals to maintain a compassionate stance toward their patients. Empathy and shared humanity was invoked as an important skill deliberately recruited to maintain compassionate responding. Self-care was also invoked as an important strategy to avoid compassion fatigue.

Further techniques in the development of compassion toward self and others comes from an art-based training program described by Bennett-Levy et al.. In their pilot project with Australia's Indigenous participants, the investigators found an improvement in compassion skills directed toward the self and others using visual art projects. The use of imagery may be more effective than traditional verbal instructions used in CFT at least in some populations.

In another application within the higher education curricula, Lundgren and Osika describe ways in which compassion could be injected in supervisor-graduate student relationship. The authors provide a framework to improve compassionate supervisor behaviors in order to foster more creative and productive relationships with students.

Fryburg et al. show evidence for a novel intervention consisting of kindness media. The authors examined the impact of pictures and images depicting kindness and compassion in hospital settings. Compared to the patients exposed to regular programming, those exposed to kindness media reported feeling more relaxed and happy after brief exposure. They were also more likely to donate their earnings from participation in the study than those exposed to regular programming. The authors suggest that witnessing others engage in compassionate action can inspire and lead to more charitable behaviors, and provide an easily implementable intervention to facilate this process.

Benevolence at the workplace is generally beneficial for the benefactor. Andersson et al. found that workers who felt able and willing to give to co-workers reported lower levels of depression, perceived stress, and emotional exhaustion in the workplace.

## Increasing Compassion Collectively

Given the benefits accrued from compassionate responding, how could we apply this knowledge to create a more compassionate world? In an empirical study, Mongrain and Shoikhedbrod illustrate how judgment and stigma pre-empt the experience and expression of compassion. Stimatized attitudes and harsh judgments toward vulnerable groups may reflect a more general societal problem. The authors suggest that broad endeavors such as public health campaigns to educate and promote greater understanding may improve empathy and compassionate responding toward marginalized groups.

As illustrated by Weng et al. we can be more inclusive and compassionate in our own research. In their “Intersectional Neuroscience Framework” the authors describe how multiple marginalized communities can be included in contemplative research. They describe individualized neuroscience methods to recognize unique brain patterns associated with internal attention states during a meditative task. In this study, brain patterns associated with attention to the breath, mind-wandering, or self-referential processing could be reliably identified for each meditor. This research presents promising methods for future research in contemplative neuroscience.

In a seminal paper on the evolutionary history of basic human drives, Gilbert describes how the acquisition of resources since the agricultural revolution 10,000 years ago has shifted human beings' fundamental drives. The ability to acquire and store resources has shifted our social mentality from one of caring and sharing (essential to the survival of hunter-gatherers) to one of “controling and holding” onto resources. This has led to the predominance of a competitive orientation in our recent evolution, concerned over the acquisition of personal wealth and power. Gilbert provides compelling arguments for the destructive outcomes of this social mentality and argues that change within our culture is urgently needed. For example, he suggests that shifts in resource distribution and societal institutions are necessary to facilitate cooperation over competition.

In the age of an interconnected world, Day et al. explain how digital technology could be harnessed to promote the greater good. While virtual networks have reduced our capacity for empathy, the authors provide ideas on how to develop technologies tapping into our potential for compassion. For example, virtual assistants could be designed to maximize personal growth. Public ownership of digital platforms could facilitate mutually-beneficial decision-making among its members. There are tremendous opportunities to enhance self-transcendance and minimize greed through the digital world and this article provides directions for future implementations.

In conclusion, this special issue provides the latest advances in our understanding of compassion, its roots, and facilitative conditions as well as its obstruction. The papers are consistent with recent views on “concious evolution” and the possibility that compassion and altruism could evolve through intentional and guided effort to shape individuals and environments. We may be at the dawn of a cultural change and a transformation in the expression of our prosocial nature where behaviors associated with self-transcendance and goodness could be promoted (Wilson, 2020). This collection of papers will hopefully inspire innovation in research and serve to inform future inter-disciplinary efforts to increase compassion in all our human affairs.

## Author Contributions

The Editorial was written by MM, and was revised by DK and JK. All authors contributed to the article and approved the submitted version.

## Conflict of Interest

The authors declare that the research was conducted in the absence of any commercial or financial relationships that could be construed as a potential conflict of interest.

## Publisher's Note

All claims expressed in this article are solely those of the authors and do not necessarily represent those of their affiliated organizations, or those of the publisher, the editors and the reviewers. Any product that may be evaluated in this article, or claim that may be made by its manufacturer, is not guaranteed or endorsed by the publisher.
